# 12-Di­methyl­amino-2,2-di­fluoro-8-phenyl-1λ^5^,3-di­aza-2λ^4^-boratri­cyclo­[7.3.0.0^3,7^]dodeca-1(12),4,6,8,10-pentaen-1-ylium

**DOI:** 10.1107/S1600536813010404

**Published:** 2013-04-24

**Authors:** Zhao-Yun Wang

**Affiliations:** a School of Chemistry and Material Science, Anhui Normal University, Wuhu, People’s Republic of China

## Abstract

In the title boron–dipyrromethene derivative, C_17_H_16_BF_2_N_3_, the benzene ring and the boron–dipyrromethene mean plane form a dihedral angle of 55.82 (8)°. In the crystal, pairs of C—H⋯F inter­actions link the mol­ecules, forming inversion dimers. Further C—H⋯F inter­actions link the dimers into a three-dimensional network.

## Related literature

For the synthesis and applications of related 4,4-di­fluoro-4-bora-3*a*,4*a*-di­aza-*s*-indacene derivatives, see: Trieflinger *et al.* (2005 ▶). For related structures, see: Jiao *et al.* (2011 ▶).
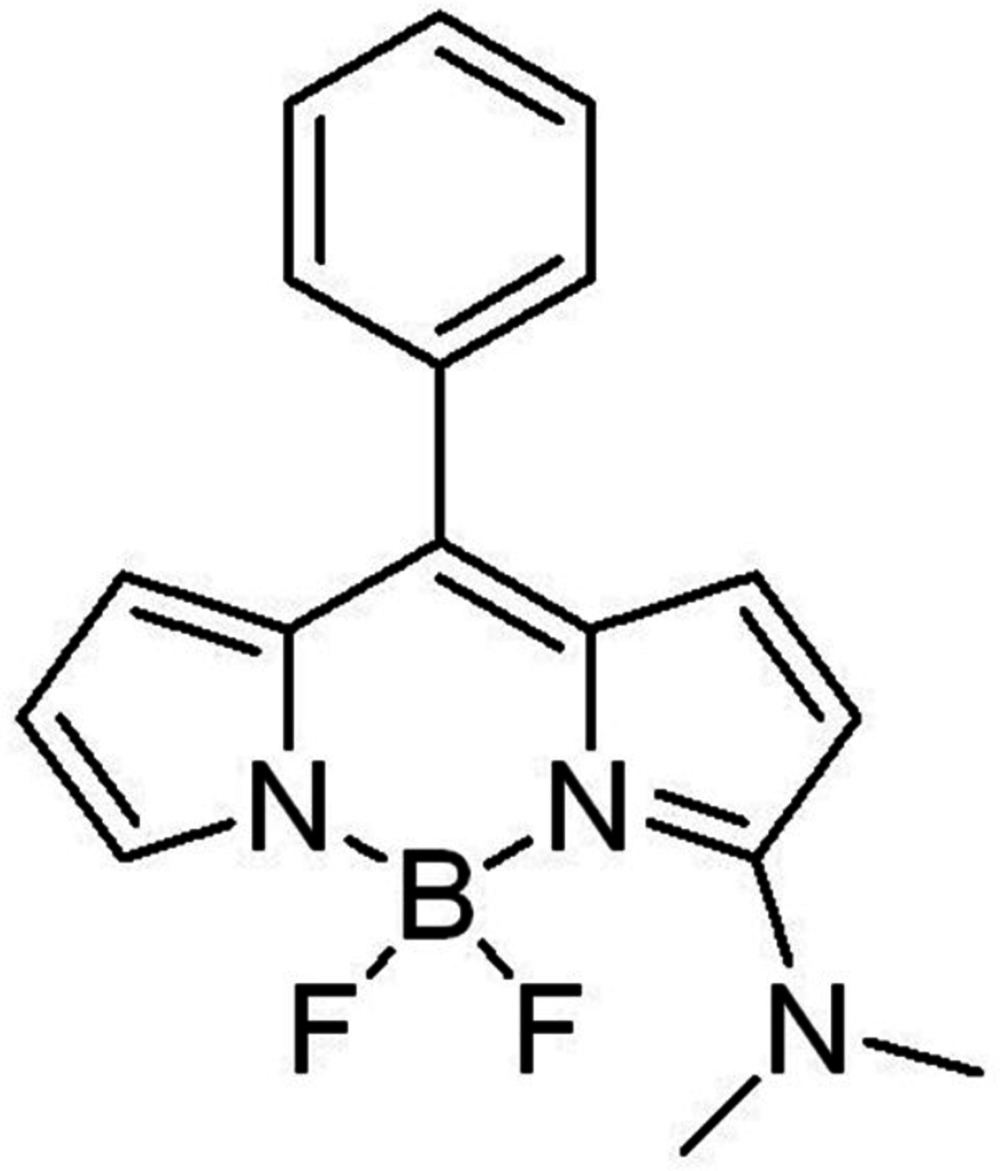


## Experimental

### 

#### Crystal data

C_17_H_16_BF_2_N_3_
*M* *_r_* = 311.14
Monoclinic, 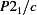

*a* = 7.8033 (6) Å
*b* = 25.524 (2) Å
*c* = 9.9776 (5) Å
β = 128.671 (4)°
*V* = 1551.5 (2) Å^3^
*Z* = 4
Mo *K*α radiation
μ = 0.10 mm^−1^
*T* = 295 K
0.30 × 0.20 × 0.20 mm

#### Data collection

Bruker SMART APEX CCD diffractometer
Absorption correction: multi-scan (*SADABS*; Sheldrick, 1996 ▶) *T*_min_ = 0.972, *T*_max_ = 0.981
11054 measured reflections
2736 independent reflections
2025 reflections with *I* > 2σ(*I*)
*R*_int_ = 0.027

#### Refinement

*R*[*F* ^2^ > 2σ(*F* ^2^)] = 0.041
*wR*(*F* ^2^) = 0.119
*S* = 1.03
2736 reflections
210 parameters
H-atom parameters constrained
Δρ_max_ = 0.16 e Å^−3^
Δρ_min_ = −0.14 e Å^−3^

### 

Data collection: *SMART* (Bruker, 2002 ▶); cell refinement: *SAINT* (Bruker, 2002 ▶); data reduction: *SAINT*; program(s) used to solve structure: *SHELXS97* (Sheldrick, 2008 ▶); program(s) used to refine structure: *SHELXL97* (Sheldrick, 2008 ▶); molecular graphics: *SHELXTL* (Sheldrick, 2008 ▶); software used to prepare material for publication: *SHELXTL*.

## Supplementary Material

Crystal structure: contains datablock(s) I, global. DOI: 10.1107/S1600536813010404/rk2400sup1.cif

Structure factors: contains datablock(s) I. DOI: 10.1107/S1600536813010404/rk2400Isup2.hkl

Supplementary material file. DOI: 10.1107/S1600536813010404/rk2400Isup3.cml

Additional supplementary materials:  crystallographic information; 3D view; checkCIF report

## Figures and Tables

**Table 1:** Hydrogen-bond geometry (Å, °)

*D*—H⋯*A*	*D*—H	H⋯*A*	*D*⋯*A*	*D*—H⋯*A*
C1—H1⋯F2^i^	0.93	2.51	3.291 (3)	142
C17—H17*C*⋯F2^ii^	0.96	2.49	3.282 (3)	140

Symmetry codes: (i) 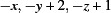; (ii) 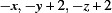.

**Fig. 1.**
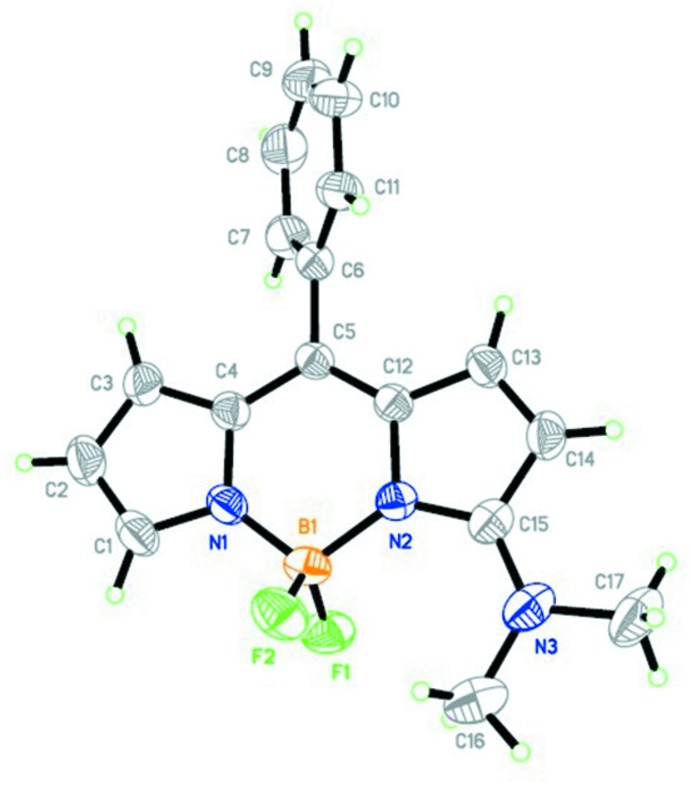
The molecular structure of title molecule showing the atom numbering scheme. Displacement ellipsoids are drawn at 30% probability level. H atoms are presented as a small spheres of arbitrary radius.

**Fig. 2.**
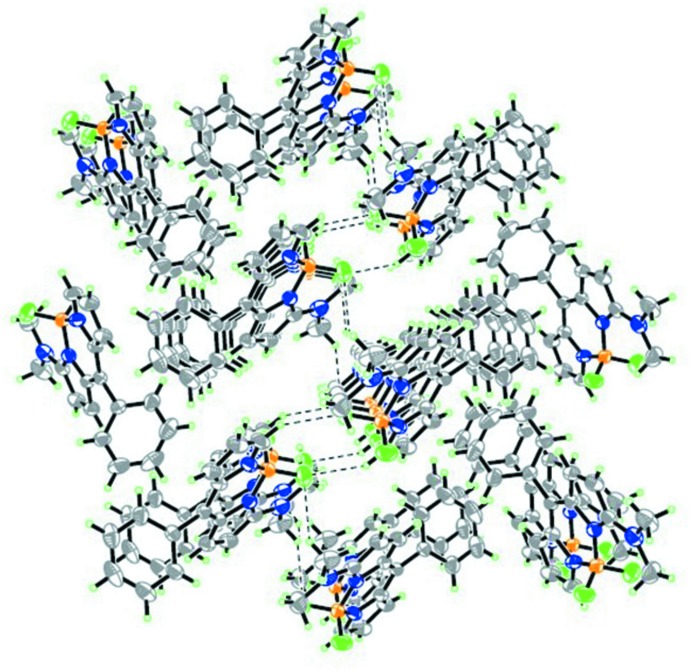
The packing diagram of the title compound. Short H···F contacts are showed by dashed lines.

## supplementary crystallographic information

### Comment

Fluorescent dyes, especially 4,4-difluoro-4-bora-3*a*,4*a*-diaza-*s*-indacene (*BODIPY*), have been led to the increased research interest in these molecules lately, *BODIPY*s have found wide applications in fluorescence labels and biomolecular sensors (Trieflinger *et al.*, 2005), due to their remarkable properties, including large molar absorption coefficient, sharp fluorescence emissions, high fluorescence quantum yields, and high photophysical stability. As part of our ongoing studies (Jiao *et al.*, 2011), we have obtained the title compound, and report its molecular structure here (Fig. 1). The bond lengths and angles are within normal ranges. By short contact C1—H1···F2^i^ = 2.512Å, molecule forms 10-members centrosymmetrical dimers. Next short contact C17—H17···F2^ii^ = 2.491Å form a three-dimensional network, which seem to be very effective in the stabilization of the crystal structure (Fig. 2). Symmetry codes: (i) -*x*, 2-*y*, 1-*z*; (ii) -*x*, 2-*y*, 2-*z*.

### Experimental

To 4,4-difluoro-8-phenyl-4-bora-3*a*,4*a*-diaza-*s*-indacene (134 mg, 0.5 mmol) in 3 ml of *DMF*. was added Potassium *tert*-butoxide (561 mg, 5 mmol). After stirring at 323 k for 3 h, the reaction was monitored by *TLC*, then the mixture was poured into water (50 ml), adjusted pH value to 7 with hydrochloric acid and extracted with CH_2_Cl_2_ (3×30 ml). Organic layers were combined, dried over Mg_2_SO_4_, and evaporated to dryness under vacuum. Purification was performed by column chromatography on silica gel using hexane / CH_2_Cl_2_ (*v*/*v*, 2:1) as eluent, from which the desired product was obtained in 51% yield (80 mg).

### Refinement

All hydrogen atoms were placed in geometrically idealized positions and constrained to ride on their parent atoms, with C—H = 0.93Å with *U*_iso_(H) = 1.2*U*_eq_(C) for aromatic H and C—H = 0.96Å with *U*_iso_(H) = 1.5*U*_eq_(C) for methyl H.

### Crystal data

**Table:**

C_17_H_16_BF_2_N_3_	*F*(000) = 648
*M**_r_* = 311.14	*D*_x_ = 1.332 Mg m^−^^3^
Monoclinic, *P*2_1_/*c*	Mo *K*α radiation, λ = 0.71073 Å
Hall symbol: -P 2ybc	Cell parameters from 4251 reflections
*a* = 7.8033 (6) Å	θ = 2.7–24.3°
*b* = 25.524 (2) Å	µ = 0.10 mm^−^^1^
*c* = 9.9776 (5) Å	*T* = 295 K
β = 128.671 (4)°	Block, colourless
*V* = 1551.5 (2) Å^3^	0.30 × 0.20 × 0.20 mm
*Z* = 4	

### Data collection

**Table:**

Bruker SMART APEX CCD diffractometer	2736 independent reflections
Radiation source: fine-focus sealed tube	2025 reflections with *I* > 2σ(*I*)
Graphite monochromator	*R*_int_ = 0.027
φ and ω scans	θ_max_ = 25.0°, θ_min_ = 1.6°
Absorption correction: multi-scan (*SADABS*; Sheldrick, 1996)	*h* = −9→8
*T*_min_ = 0.972, *T*_max_ = 0.981	*k* = −30→30
11054 measured reflections	*l* = −11→11

### Refinement

**Table:**

Refinement on *F*^2^	Primary atom site location: structure-invariant direct methods
Least-squares matrix: full	Secondary atom site location: difference Fourier map
*R*[*F*^2^ > 2σ(*F*^2^)] = 0.041	Hydrogen site location: inferred from neighbouring sites
*wR*(*F*^2^) = 0.119	H-atom parameters constrained
*S* = 1.03	*w* = 1/[σ^2^(*F*_o_^2^) + (0.0582*P*)^2^ + 0.2638*P*] where *P* = (*F*_o_^2^ + 2*F*_c_^2^)/3
2736 reflections	(Δ/σ)_max_ = 0.005
210 parameters	Δρ_max_ = 0.16 e Å^−^^3^
0 restraints	Δρ_min_ = −0.14 e Å^−^^3^

### Special details

**Table:**

Geometry. All s.u.'s (except the s.u. in the dihedral angle between two l.s. planes) are estimated using the full covariance matrix. The cell s.u.'s are taken into account individually in the estimation of s.u.'s in distances, angles and torsion angles; correlations between s.u.'s in cell parameters are only used when they are defined by crystal symmetry. An approximate (isotropic) treatment of cell s.u.'s is used for estimating s.u.'s involving l.s. planes.
Refinement. Refinement of *F*^2^ against ALL reflections. The weighted *R*-factor *wR* and goodness of fit *S* are based on *F*^2^, conventional *R*-factors *R* are based on *F*, with *F* set to zero for negative *F*^2^. The threshold expression of *F*^2^ > σ(*F*^2^) is used only for calculating *R*-factors(gt) *etc*. and is not relevant to the choice of reflections for refinement. *R*-factors based on *F*^2^ are statistically about twice as large as those based on *F*, and *R*-factors based on ALL data will be even larger.

### Fractional atomic coordinates and isotropic or equivalent isotropic displacement parameters (Å^2^)

**Table:**

	*x*	*y*	*z*	*U*_iso_*/*U*_eq_	
F1	−0.28223 (17)	0.91338 (5)	0.54009 (14)	0.0842 (4)	
F2	−0.0433 (2)	0.98113 (4)	0.67169 (16)	0.0884 (4)	
N1	0.0902 (2)	0.90034 (6)	0.65707 (18)	0.0593 (4)	
N2	−0.0100 (2)	0.91017 (5)	0.84906 (17)	0.0543 (4)	
N3	−0.2640 (3)	0.95619 (7)	0.8700 (2)	0.0779 (5)	
C1	0.1083 (4)	0.91003 (9)	0.5324 (3)	0.0760 (6)	
H1	0.0217	0.9335	0.4423	0.091*	
C2	0.2720 (4)	0.88033 (10)	0.5591 (3)	0.0821 (6)	
H2	0.3155	0.8798	0.4910	0.098*	
C3	0.3621 (3)	0.85096 (8)	0.7062 (2)	0.0685 (5)	
H3	0.4772	0.8272	0.7554	0.082*	
C4	0.2477 (3)	0.86380 (7)	0.7662 (2)	0.0552 (4)	
C5	0.2796 (3)	0.84981 (6)	0.9186 (2)	0.0510 (4)	
C6	0.4562 (3)	0.81223 (7)	1.0379 (2)	0.0563 (4)	
C7	0.4630 (4)	0.76322 (7)	0.9807 (3)	0.0757 (6)	
H7	0.3519	0.7533	0.8678	0.091*	
C8	0.6331 (5)	0.72935 (9)	1.0903 (4)	0.0968 (8)	
H8	0.6366	0.6966	1.0513	0.116*	
C9	0.7971 (5)	0.74379 (12)	1.2564 (4)	0.1039 (9)	
H9	0.9126	0.7209	1.3295	0.125*	
C10	0.7929 (4)	0.79204 (11)	1.3165 (3)	0.0923 (7)	
H10	0.9040	0.8015	1.4300	0.111*	
C11	0.6232 (3)	0.82612 (8)	1.2073 (2)	0.0696 (5)	
H11	0.6203	0.8587	1.2474	0.084*	
C12	0.1544 (3)	0.87210 (6)	0.9555 (2)	0.0512 (4)	
C13	0.1508 (3)	0.86084 (7)	1.0927 (2)	0.0629 (5)	
H13	0.2419	0.8372	1.1811	0.075*	
C14	−0.0056 (3)	0.88989 (8)	1.0739 (3)	0.0705 (5)	
H14	−0.0427	0.8897	1.1462	0.085*	
C15	−0.1061 (3)	0.92141 (7)	0.9228 (2)	0.0621 (5)	
C16	−0.3699 (4)	0.99063 (10)	0.7212 (4)	0.1032 (8)	
H16A	−0.2601	1.0099	0.7266	0.155*	
H16B	−0.4650	1.0146	0.7208	0.155*	
H16C	−0.4545	0.9700	0.6181	0.155*	
C17	−0.3488 (4)	0.96200 (12)	0.9644 (3)	0.1063 (9)	
H17A	−0.3107	0.9317	1.0349	0.159*	
H17B	−0.5057	0.9655	0.8847	0.159*	
H17C	−0.2860	0.9926	1.0356	0.159*	
B1	−0.0669 (3)	0.92741 (8)	0.6751 (3)	0.0601 (5)	

### Atomic displacement parameters (Å^2^)

**Table:**

	*U*^11^	*U*^22^	*U*^33^	*U*^12^	*U*^13^	*U*^23^
F1	0.0555 (6)	0.1025 (9)	0.0600 (7)	0.0046 (6)	0.0193 (6)	−0.0029 (6)
F2	0.1155 (10)	0.0568 (7)	0.0922 (9)	0.0042 (6)	0.0646 (8)	0.0128 (6)
N1	0.0582 (8)	0.0667 (9)	0.0467 (8)	−0.0010 (7)	0.0297 (7)	0.0060 (7)
N2	0.0504 (8)	0.0549 (8)	0.0527 (8)	0.0015 (6)	0.0299 (7)	−0.0012 (6)
N3	0.0586 (9)	0.0861 (12)	0.0789 (12)	0.0042 (9)	0.0379 (9)	−0.0200 (9)
C1	0.0825 (14)	0.0895 (14)	0.0521 (11)	−0.0032 (12)	0.0402 (11)	0.0119 (10)
C2	0.0860 (15)	0.1096 (17)	0.0647 (13)	−0.0059 (13)	0.0539 (12)	0.0010 (12)
C3	0.0648 (11)	0.0866 (13)	0.0593 (11)	0.0004 (10)	0.0414 (10)	−0.0020 (10)
C4	0.0523 (9)	0.0622 (10)	0.0466 (9)	−0.0033 (8)	0.0287 (8)	−0.0012 (8)
C5	0.0501 (9)	0.0512 (9)	0.0462 (9)	−0.0036 (7)	0.0274 (8)	−0.0015 (7)
C6	0.0579 (10)	0.0579 (10)	0.0564 (11)	0.0046 (8)	0.0373 (9)	0.0069 (8)
C7	0.0912 (15)	0.0622 (11)	0.0837 (14)	0.0110 (11)	0.0595 (13)	0.0051 (10)
C8	0.122 (2)	0.0736 (14)	0.128 (2)	0.0338 (15)	0.094 (2)	0.0272 (15)
C9	0.0907 (18)	0.109 (2)	0.126 (2)	0.0468 (16)	0.0742 (19)	0.0593 (18)
C10	0.0657 (13)	0.1115 (19)	0.0761 (15)	0.0170 (13)	0.0327 (12)	0.0309 (14)
C11	0.0610 (11)	0.0743 (12)	0.0601 (12)	0.0052 (10)	0.0312 (10)	0.0095 (10)
C12	0.0515 (9)	0.0515 (9)	0.0472 (9)	−0.0021 (7)	0.0292 (8)	−0.0016 (7)
C13	0.0655 (11)	0.0714 (11)	0.0520 (10)	−0.0016 (9)	0.0368 (9)	0.0004 (9)
C14	0.0677 (12)	0.0925 (14)	0.0606 (12)	−0.0045 (11)	0.0446 (10)	−0.0108 (10)
C15	0.0522 (10)	0.0667 (11)	0.0604 (11)	−0.0061 (9)	0.0317 (9)	−0.0164 (9)
C16	0.0863 (16)	0.0873 (16)	0.119 (2)	0.0278 (14)	0.0557 (16)	0.0073 (15)
C17	0.0768 (15)	0.141 (2)	0.1026 (19)	0.0081 (15)	0.0569 (15)	−0.0398 (16)
B1	0.0562 (12)	0.0545 (11)	0.0519 (12)	−0.0006 (9)	0.0253 (10)	0.0022 (9)

### Geometric parameters (Å, º)

**Table:**

F1—B1	1.392 (2)	C6—C7	1.390 (3)
F2—B1	1.387 (2)	C7—C8	1.375 (3)
N1—C1	1.359 (2)	C7—H7	0.9300
N1—C4	1.376 (2)	C8—C9	1.368 (4)
N1—B1	1.513 (3)	C8—H8	0.9300
N2—C15	1.369 (2)	C9—C10	1.379 (4)
N2—C12	1.420 (2)	C9—H9	0.9300
N2—B1	1.562 (3)	C10—C11	1.376 (3)
N3—C15	1.331 (2)	C10—H10	0.9300
N3—C17	1.458 (3)	C11—H11	0.9300
N3—C16	1.458 (3)	C12—C13	1.416 (2)
C1—C2	1.361 (3)	C13—C14	1.337 (3)
C1—H1	0.9300	C13—H13	0.9300
C2—C3	1.386 (3)	C14—C15	1.435 (3)
C2—H2	0.9300	C14—H14	0.9300
C3—C4	1.388 (3)	C16—H16A	0.9600
C3—H3	0.9300	C16—H16B	0.9600
C4—C5	1.425 (2)	C16—H16C	0.9600
C5—C12	1.365 (2)	C17—H17A	0.9600
C5—C6	1.479 (2)	C17—H17B	0.9600
C6—C11	1.389 (3)	C17—H17C	0.9600
			
C1—N1—C4	107.13 (16)	C11—C10—C9	119.5 (2)
C1—N1—B1	125.53 (16)	C11—C10—H10	120.2
C4—N1—B1	127.26 (14)	C9—C10—H10	120.2
C15—N2—C12	106.50 (14)	C10—C11—C6	120.6 (2)
C15—N2—B1	131.83 (15)	C10—C11—H11	119.7
C12—N2—B1	121.37 (14)	C6—C11—H11	119.7
C15—N3—C17	119.6 (2)	C5—C12—C13	128.79 (16)
C15—N3—C16	126.89 (19)	C5—C12—N2	123.22 (15)
C17—N3—C16	113.53 (19)	C13—C12—N2	107.89 (15)
N1—C1—C2	109.95 (18)	C14—C13—C12	108.66 (17)
N1—C1—H1	125.0	C14—C13—H13	125.7
C2—C1—H1	125.0	C12—C13—H13	125.7
C1—C2—C3	107.53 (18)	C13—C14—C15	108.04 (17)
C1—C2—H2	126.2	C13—C14—H14	126.0
C3—C2—H2	126.2	C15—C14—H14	126.0
C2—C3—C4	106.95 (19)	N3—C15—N2	127.54 (18)
C2—C3—H3	126.5	N3—C15—C14	123.55 (18)
C4—C3—H3	126.5	N2—C15—C14	108.89 (16)
N1—C4—C3	108.44 (16)	N3—C16—H16A	109.5
N1—C4—C5	119.19 (15)	N3—C16—H16B	109.5
C3—C4—C5	132.08 (17)	H16A—C16—H16B	109.5
C12—C5—C4	120.46 (15)	N3—C16—H16C	109.5
C12—C5—C6	121.14 (15)	H16A—C16—H16C	109.5
C4—C5—C6	118.36 (15)	H16B—C16—H16C	109.5
C11—C6—C7	118.84 (18)	N3—C17—H17A	109.5
C11—C6—C5	120.53 (16)	N3—C17—H17B	109.5
C7—C6—C5	120.58 (17)	H17A—C17—H17B	109.5
C8—C7—C6	120.3 (2)	N3—C17—H17C	109.5
C8—C7—H7	119.8	H17A—C17—H17C	109.5
C6—C7—H7	119.8	H17B—C17—H17C	109.5
C9—C8—C7	120.1 (2)	F2—B1—F1	109.21 (15)
C9—C8—H8	119.9	F2—B1—N1	108.58 (16)
C7—C8—H8	119.9	F1—B1—N1	110.18 (16)
C8—C9—C10	120.6 (2)	F2—B1—N2	110.87 (15)
C8—C9—H9	119.7	F1—B1—N2	109.63 (16)
C10—C9—H9	119.7	N1—B1—N2	108.35 (14)
			
C4—N1—C1—C2	0.4 (2)	C15—N2—C12—C5	177.30 (15)
B1—N1—C1—C2	177.39 (18)	B1—N2—C12—C5	2.9 (2)
N1—C1—C2—C3	−0.4 (3)	C15—N2—C12—C13	0.65 (18)
C1—C2—C3—C4	0.2 (2)	B1—N2—C12—C13	−173.74 (15)
C1—N1—C4—C3	−0.3 (2)	C5—C12—C13—C14	−176.43 (17)
B1—N1—C4—C3	−177.21 (16)	N2—C12—C13—C14	0.0 (2)
C1—N1—C4—C5	174.28 (16)	C12—C13—C14—C15	−0.6 (2)
B1—N1—C4—C5	−2.6 (3)	C17—N3—C15—N2	178.89 (18)
C2—C3—C4—N1	0.1 (2)	C16—N3—C15—N2	−0.4 (3)
C2—C3—C4—C5	−173.55 (19)	C17—N3—C15—C14	−2.5 (3)
N1—C4—C5—C12	0.4 (2)	C16—N3—C15—C14	178.2 (2)
C3—C4—C5—C12	173.53 (18)	C12—N2—C15—N3	177.73 (17)
N1—C4—C5—C6	−177.34 (15)	B1—N2—C15—N3	−8.7 (3)
C3—C4—C5—C6	−4.2 (3)	C12—N2—C15—C14	−1.01 (18)
C12—C5—C6—C11	−56.0 (2)	B1—N2—C15—C14	172.56 (17)
C4—C5—C6—C11	121.79 (18)	C13—C14—C15—N3	−177.78 (17)
C12—C5—C6—C7	126.68 (19)	C13—C14—C15—N2	1.0 (2)
C4—C5—C6—C7	−55.6 (2)	C1—N1—B1—F2	−51.6 (2)
C11—C6—C7—C8	−0.4 (3)	C4—N1—B1—F2	124.74 (18)
C5—C6—C7—C8	177.02 (19)	C1—N1—B1—F1	68.0 (2)
C6—C7—C8—C9	−0.1 (4)	C4—N1—B1—F1	−115.69 (19)
C7—C8—C9—C10	0.7 (4)	C1—N1—B1—N2	−172.08 (16)
C8—C9—C10—C11	−0.8 (4)	C4—N1—B1—N2	4.2 (2)
C9—C10—C11—C6	0.3 (3)	C15—N2—B1—F2	63.9 (2)
C7—C6—C11—C10	0.3 (3)	C12—N2—B1—F2	−123.27 (17)
C5—C6—C11—C10	−177.12 (18)	C15—N2—B1—F1	−56.7 (2)
C4—C5—C12—C13	175.20 (16)	C12—N2—B1—F1	116.07 (17)
C6—C5—C12—C13	−7.1 (3)	C15—N2—B1—N1	−176.99 (16)
C4—C5—C12—N2	−0.7 (2)	C12—N2—B1—N1	−4.2 (2)
C6—C5—C12—N2	176.99 (15)		

### Hydrogen-bond geometry (Å, º)

**Table:**

*D*—H···*A*	*D*—H	H···*A*	*D*···*A*	*D*—H···*A*
C1—H1···F2^i^	0.93	2.51	3.291 (3)	142
C17—H17*C*···F2^ii^	0.96	2.49	3.282 (3)	140

Symmetry codes: (i) −*x*, −*y*+2, −*z*+1; (ii) −*x*, −*y*+2, −*z*+2.
